# Female rats are resilient to the behavioral effects of maternal separation stress and exhibit stress-induced neurogenesis

**DOI:** 10.1016/j.heliyon.2020.e04753

**Published:** 2020-08-21

**Authors:** Yan Jun Lee, Amelia S. Koe, Archana Ashokan, Rupshi Mitra

**Affiliations:** School of Biological Sciences, Nanyang Technological University, 60 Nanyang Drive 637551, Singapore

**Keywords:** Amygdala, Anxiety, Gender, Hypertrophy, Neurogenesis, Behavioral neuroscience, Nervous system, Cellular neuroscience, Early-life

## Abstract

Early-life stress causes anxiogenesis and sensitivity of stress endocrine axis, facilitated by changes in the basolateral amygdala and hippocampal neurogenesis. In this report, we examined if male-like relationship between early-life stress and anxiety was recapitulated in female rats, along with related neurobiological substrates of the amygdala and the hippocampus. Maternal separation, a paradigm consistently utilized in male rats in most previously published scripts, did not cause similar behavioral consequences in females. Maternal separation caused an increase in adult hippocampal neurogenesis in females without causing substantial differences in dendritic arbors of the basolateral amygdala. Thus, female rats displayed remarkable resilience in the emotional consequences of early-life stress.

## Teaser

Amygdala, Anxiety, Gender, Hypertrophy, Neurogenesis, Behavioral neuroscience, Nervous system, Cellular neuroscience, Neuroanatomy, Mental disorder.

## 1 Introduction

The quality and quantity of interactions with the mother have long-term consequences for the emotional development of the offspring. Reduced maternal care or periods of maternal absence leads to a higher incidence of psychiatric disorders [1] and lower life expectancy in humans [2]. This phenomenon has also been repeatedly modeled in rats and mice using paradigms of maternal separation, whereby dams are intermittently separated from the pups during the pre-weaning stage [3]. These studies show that maternal separation causes a maladaptive stress response in the exposed pups as they reach adulthood [4, 5]. The reported effects also include an increase in anxiety-like behaviors and a decrease in adult neurogenesis [6, 7]. Maternal separation also causes sustained plasticity within the hippocampus and the amygdala [8, 9], brain regions that form crucial parts of brain circuits regulating stress endocrine response. This is not surprising because the separation coincides with critical periods of brain development where brain regions exerting central control on stress responses are maturing and are thus open to ontogenic shifts due to changes in the maternal environment [10].

Gender is an essential factor in understanding the effects of early-life stress on adult behaviors. The development of the brain during early life responds to the sex-dependent hormonal milieu of developing offspring. Moreover, sex hormones in adulthood can modify the brain and behavior due to maternal separation. Nevertheless, majority of studies about maternal separation sample male animals. These findings, focused as they are on a circumscribed and non-representative gender distribution, might not explain the effects of early-life stress on the female neurobiology and the corresponding behavior. For example, the maternal separation paradigms that lead to persistent anxiety in male rats does not cause anxiety in females [11, 12], a phenomenon in contrast to higher anxiety in female humans [13, 14]. Similarly, the same perinatal stressors that lead to reduced proliferation and survival of new hippocampal neurons in males do not cause appreciable loss of neurogenesis in females [15, 16]. In the same vein, chronic stressors during adulthood cause a long-lasting dendritic expansion in principal neurons of the basolateral amygdala (BLA) in males [17]. Yet, amygdala neural hypertrophy is reversed when a chimeric receptor is used to bind to glucocorticoids but drive genomic response through estrogen-responsive elements [18]. Thus, gender is likely to be an important variable when the long-term effects of maternal separation are discussed and interpreted. Hence, we studied the female-specific effects of prior maternal separation on anxiety, hippocampal neurogenesis, and BLA plasticity in the present report, using three separate cohorts of female animals for each investigation.

## 2 Materials and methods

### 2.1 Animals and treatments

All animal experiments were reviewed and approved by the Nanyang Technological University's Institutional Animal Care and Use Committee. We obtained Wistar rats (InVivos, Singapore) and housed them at Nanyang Technological University vivarium (12h light-dark cycle with lights on at 0700h). Females were mated, and the males were removed upon confirmation of the pregnancy (through manual palpation of the lower abdomen). A total of nine litters were used in the study, randomly allocated to experimental treatments. Litters were culled to 12 pups per mother when necessary (postnatal day 2 or PND 2), and randomly assigned to experimental groups (AFR: animal facility rearing; or, MS: Maternal separation). There was no difference in age of mothers or the process of breeding between animals. AFR litters were left undisturbed except for cage changes on PND 2, 9, and 14. In addition to cage changes, MS litters were subjected to maternal separation for 180 min a day from PND 2 to PND 14, starting at 0900h. Both groups were weaned on PND 21.

### 2.2 Anxiety

Experimenters were blind to experimental treatments during behavioral experiments and subsequent analysis. Anxiety-like behavior was tested using home-cage emergence assay (PND 70) and elevated plus-maze (PND 74) [9,19]. Elevated plus-maze consisted of two open (75 × 11 cm^2,^ 5 cm high) and two enclosed arms (75 × 11 cm^2,^ 26 cm high), elevated 60cm from ground. Open arms were dimly lit at 6 lux. Exploration in the open arms, relative to enclosed arms, was quantified (trial duration = 5 min). Entry into an arm was defined as all four paws along with the base of the tail of the animal in a single arm. Entries made and time spent in the open and the enclosed arm were measured. Open arm entries and time was normalized to total entries and total trial duration. Home cage emergence assay was conducted by placing the home cage of the animals 10cm apart from a novel standard cage filled with unsoiled bedding (dim light = 3–4 lux in both cages). Lids were removed from both the cages. A metal grid was placed against one of the edges of the home cage, leading into the novel cage. Latency to leave the home cage was measured, which was defined as all four paws of the animal on the metal grid.

### 2.3 Dendritic parameters of basolateral amygdala neurons

Animals were sacrificed on PND 65 by rapid decapitation, and freshly harvested brain tissue was processed for Golgi-Cox staining using commercially available reagents (FD Technologies, USA). Brains were cryoprotected by equilibration with 30% sucrose (w/v) in buffered saline and subsequently sectioned in the coronal plane at a thickness of 100 μm in a cryotome (Leica CM3050-S, Leica Biosystems). Sections spanning BLA (Bregma -1.88 to -3.80 mm) were collected on glass slides, dehydrated in an ascending series of ethyl alcohol, cleared in xylene and coverslipped with Permount (Fisher Scientific). Camera lucida drawings of individual neurons were obtained at 400x (Olympus BX43, Japan) using previously published selection criteria [9]. Custom designed macros embedded in ‘ImageJ’ software were then used for morphometric analyses of digitized images. Dendritic length and the total number of branch points for principal neurons of the BLA were quantified. We specifically chose to investigate amygdalar dendrities because of earlier work showing its importance for stress-induced anxiogenesis in males [9, 17]. Multiple neurons were analyzed per animal, and the mean of neurons within an individual animal was used as the relevant endpoint for within-group analysis (neurons/animal: 5.5 ± 0.4 for AFR and 5.7 ± 0.5 for MS). Number of neurons analyzed per animal was not different between groups (t_22_ = 0.310, *p* = 0.760).

### 2.4 Neurogenesis

Cells proliferating between PND 23 to 26 were labeled by daily injection of BrdU (5-bromo-2-deoxyuridine, 100 mg/kg body weight, *i.p.*; Acros Organics). Rats were sacrificed on PND 56 by transcardial perfusion of buffered saline followed by 4% paraformaldehyde. Harvested brains were cryoprotected by equilibration with 30% sucrose (w/v) in buffered saline and subsequently sectioned in the coronal plane at a thickness of 40 μm in cryotome. Coronal sections were obtained spanning dorsal and ventral hippocampus (dorsal: Bregma -2.28 to -4.36 mm; ventral: -4.44 to -6.30 mm). Every 6^th^ section was stained for visualization of BrdU positive cells, using sequential incubation with anti-BrdU primary antibody (Santa Cruz; diluted 1:1000, overnight), secondary biotinylated horse anti-mouse antibody (Vector Laboratories; 1:200, 30 min), avidin bound horseradish peroxidase and later chromogenic development using diaminobenzidine. Sections were dehydrated in an ascending series of ethyl alcohol, cleared in xylene, and coverslipped with Permount. BrdU-positive cells present in the dentate gyrus in the dorsal and ventral hippocampus were manually counted at 400X optical magnification (dorsal: bregma -2.28mm to -4.36mm; ventral: -4.44mm to -6.30mm). Only medium and large stained nuclei in the dentate gyrus were quantified. At the same time, rod-shaped endothelial-like and small BrdU-positive cells, suggestive of glial cells and/or precursors, were excluded from the analysis. Amygdala does not show substantial neurogenesis in adulthood and was not analyzed in the present study.

### 2.5 Statistics

Each dataset was tested for the presence of outliers using the ROUT method with a maximum false discovery rate set at 1% [20]. Statistical outliers were absent from data for all endpoints. The normality of data was confirmed using the Shapiro-Wilk test. Inter-group differences for behavioral and dendritic parameters were analyzed using an independent sample t-test. An analysis of variance was used for analyzing data for neurogenesis, with dentate gyrus of ventral/dorsal hippocampus serving as within-subject and experimental treatment serving as a between-subject source of variance. Data are depicted in figures as mean and SEM, along with raw values for all animals (see Supplementary Data 1 for numerical values).

## 3 Results

### 3.1 Anxiety

**Figure 1:**
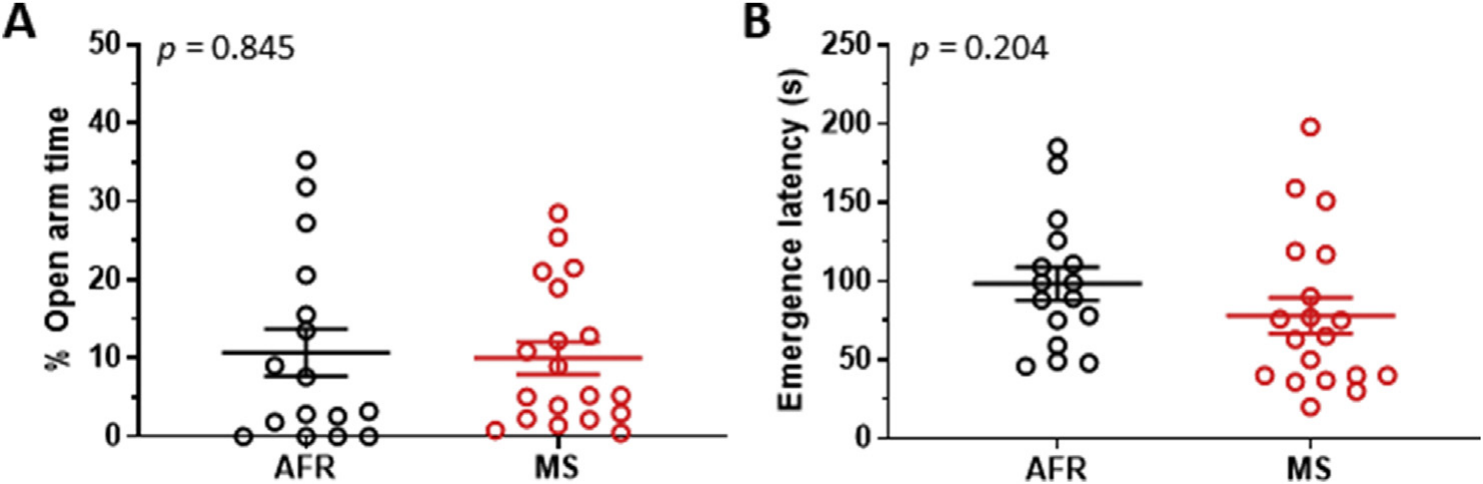
The maternal separation did not cause statistically significant anxiogenesis in female rats, as evidenced by percentage time spent in open-arms of an elevated plus-maze (panel A, relative to trial duration) and emergence latency from the home cage (panel B). AFR = animal facility rearing (n = 16 animals, black circles). MS = maternal separation (n = 19 animals, red circles). Mean, and SeM is depicted using horizontal lines.

Number of entries made into the enclosed arm was not different between AFR and MS (t_33_ < 1, *p* = 0.570). An unpaired t-test confirmed lack of statistical significance for percentage open arm entries (t_33_ = 1.58, *p* = 0.124). Lack of inter-group differences was also evident in terms of percentage time spent in the open arms (Figure 1A; t_33_ = 0.20, *p* = 0.845). Similarly, inter-group differences did not reach statistical significance for number of head dips made on the plus-maze (t_33_ = 1.25, *p* = 0.220). Thus, maternal separation did not cause anxiogenesis in female rats.

The home cage emergence assay confirmed lack of significant anxiogenesis by MS. AFR and MS did not exhibit significant differences in terms of latency to emerge from the home cage (Figure 1B; t_33_ = 1.29, *p* = 0.204).

### 3.2 Dendritic parameters of basolateral amygdala neurons

**Figure 2:**
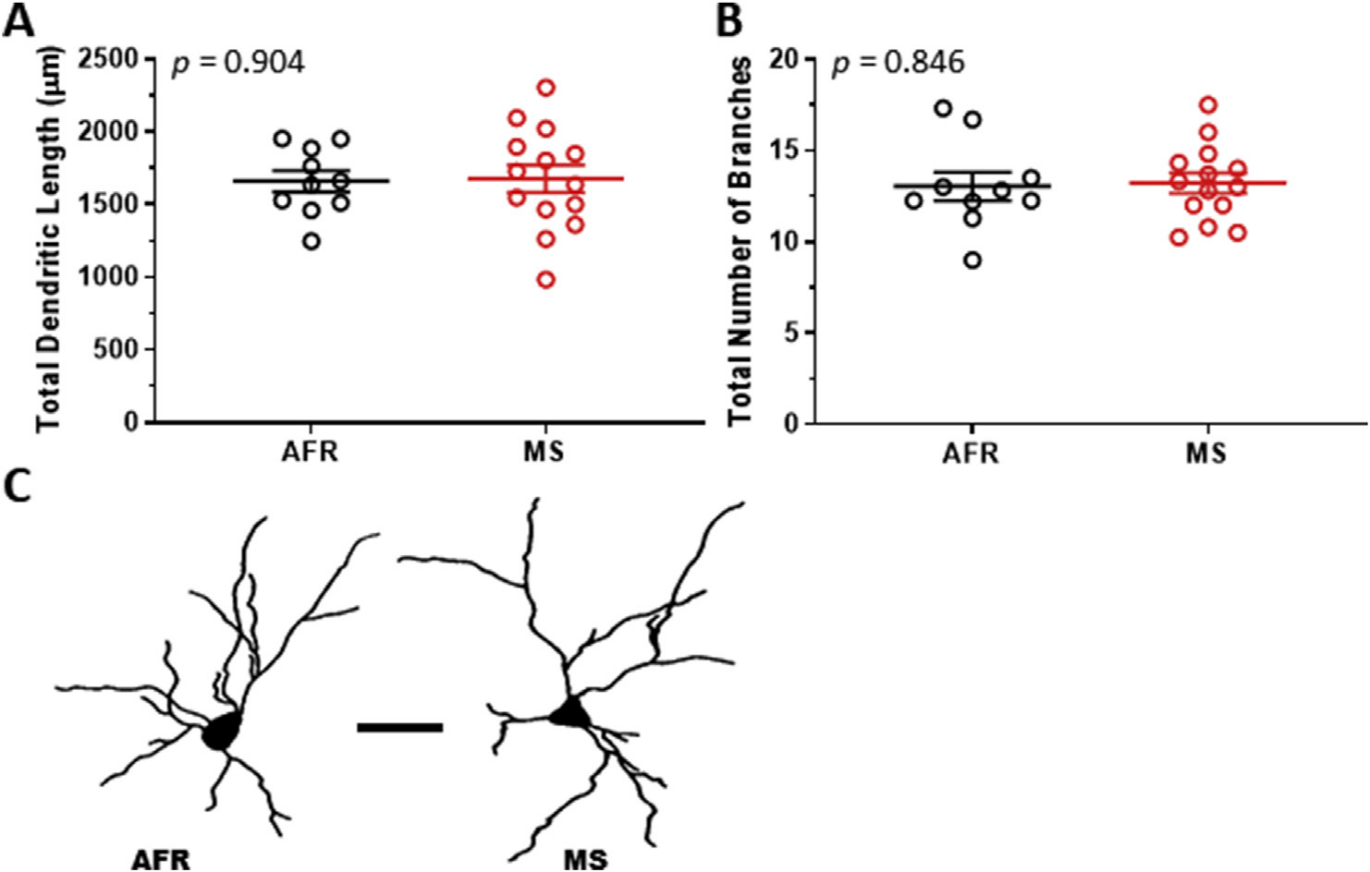
The maternal separation did not cause statistically significant dendritic expansion of principal basolateral amygdala neurons in female rats, as evidenced by total dendritic length (panel A) and the total number of branch points (panel B). AFR = animal facility rearing (n = 10 animals, black circles). MS = maternal separation (n = 14 animals, red circles). Representative dendritic traces are depicted (panel C). Scale bar = 50 μm.

MS did not cause significant changes in total dendritic length of principal BLA neurons (Figure 2A; t_22_ = 0.12, *p* = 0.904; *r* = 0.03). Similarly, total number of branch points was not significantly different between the groups (Figure 2B; t_22_ = 0.20, *p* = 0.846; *r* = 0.04). Thus, maternal separation did not cause dendritic expansion of BLA neurons in female rats (representative neurons in Figure 2C).

### 3.3 Neurogenesis

**Figure 3:**
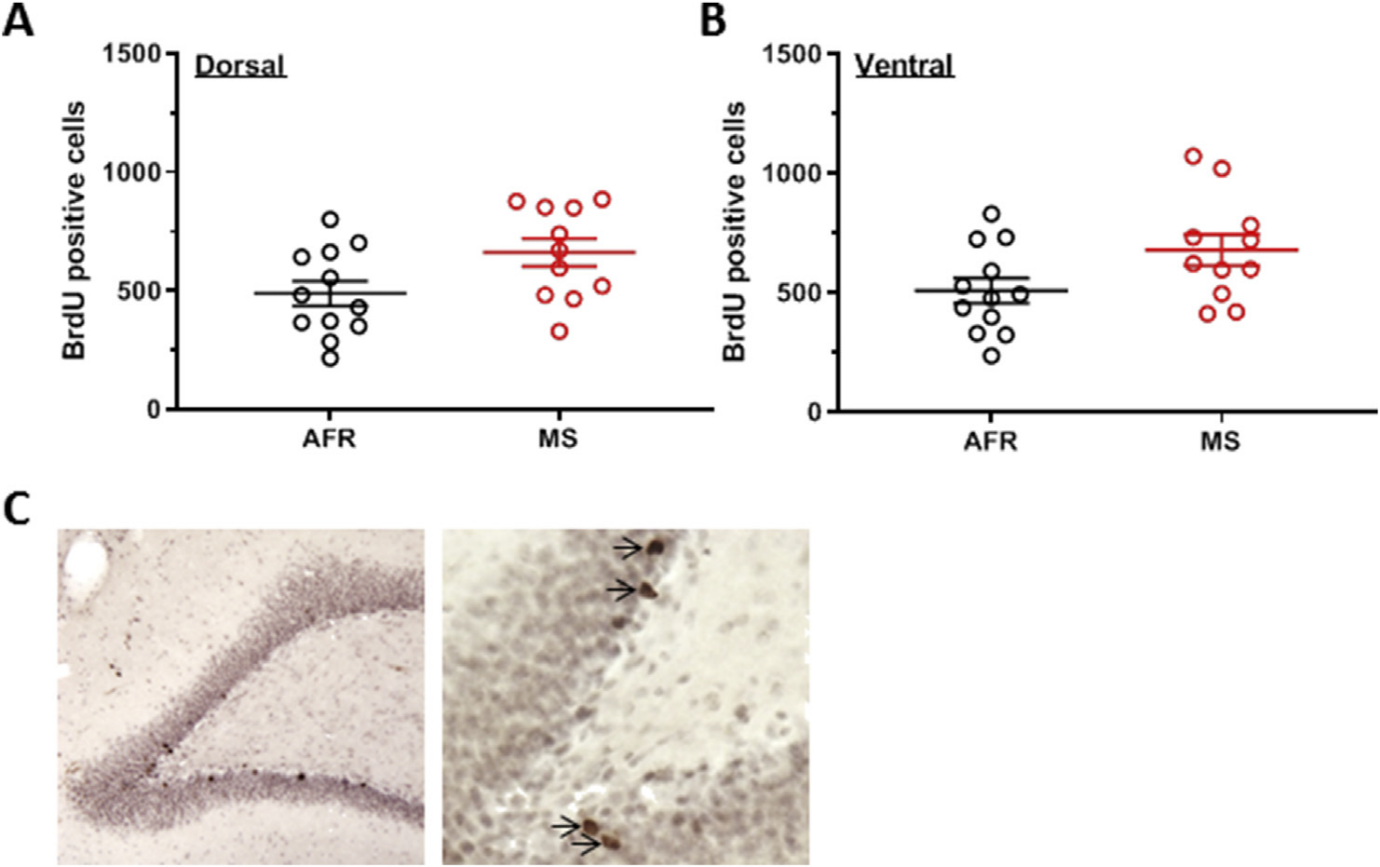
Maternal separation increased the number of neurons that were proliferating at post-natal days 23 through 26 and survived till post-natal day 56. This was evident in the main effect of experimental groups in an analysis of variance. Dentate gyrus of the dorsal hippocampus (panel A) and ventral dentate gyrus (panel B). AFR = animal facility rearing (n = 12 animals, black circles). MS = maternal separation (n = 11 animals, red circles). *p* ≤ 0.05, unpaired student's sample t-test. Representative photomicrographs of BrdU staining in dentate gyrus are depicted (panel C, at 100X and 400X optical magnification, left and right, respectively). BrdU positive cell bodies are depicted with arrows.

An analysis of variance was conducted with AFR or MS as a between-subject source of variance and dorsal or ventral hippocampal aspect of the dentate gyrus as a within-subject source of variance. Experimental treatment significantly affected dentate gyrus neurogenesis (main effect: F_1,21_ = 5.11, *p* = 0.035). Neurogenesis was not significantly different between dentate gyrus in dorsal and ventral hippocampus (main effect: F_1,21_ = 0.39, *p* = 0.540). Similarly, interaction between experimental treatment and brain regions did not reach statistical significance (F_1,21_ = 0.0005, *p* = 0.982). Thus, maternal separation increased neurogenesis in the dentate gyrus (representative images in Figure 3C).

## 4 Discussion

Data described in three preceding paragraphs show that maternal separation does not lead to anxiogenesis or BLA reorganization in adulthood in female rats. Moreover, maternal separation leads to an atypical increase in the generation of new cells within hippocampal, in contrast to earlier reports using the male rats [6, 7]. Thus, the effects of maternal separation on adult behavior are dependent on the sex of the test animals, with females exhibiting relative protection from the adverse effects.

Indicative gender dimorphism in the anxiogenesis is interesting because female humans are diagnosed with anxiety disorders at much higher rates than males [13, 14]. Rodents also exhibit gender dimorphism in anxiety, albeit in a divergent manner to humans. Data presented here show that female rats are protected from the effects of maternal separation, demonstrating a lack of anxiogenesis and BLA neuronal hypertrophy along with increased dentate gyrus neurogenesis. This is in agreement with earlier reports showing a lack of anxiogenesis in female rodents after exposure to chronic stressors in adulthood [11, 12, 21]. This protection likely reflects the protective role of ovarian steroids and buffering of stress effects within the BLA by estrogens [22]. Adrenal glucocorticoids secreted during stress engage their cognate receptors within the amygdala and initiate a transcriptional response resulting in long-term plasticity. Disruption of this signaling by estrogen can protect the amygdala from stress-induced plasticity and lead to reduced anxiety [18]. Congruently, human females experience a higher incidence of anxiety during the post-menopausal phase when estrogens are no longer available to the brain in abundance [23]. These observations support the notion that the distinct hormonal milieu of females create emotional resilience to stressful experiences and can protect against adverse effects of maternal separation.

Estrogen also facilitates neurogenesis. This is supported by the reduction of neurogenesis in ovariectomized rats and by the increase in hippocampal neurogenesis after treatment with an estrogen receptor agonist [24, 25]. Chronic stress during adulthood leads to sexually dimorphic effects on neurogenesis. For example, exposure to predator odor causes reduced neurogenesis in males but not female rodents [26]. It is noteworthy here that the effects of maternal separation on hippocampal neurogenesis are dependent upon the age when the proliferation and survival are measured. In males exposed to maternal separation, hippocampal neurogenesis presents with a biphasic response with an increase in adolescence (PND 21), no effects in adulthood (2 months), and a decrease in the middle age (15 months) [27]. Such an increase in plasticity during the perinatal period with eventual compensation in later life is a common feature of stress-induced reprogramming. For example, prenatal stress or exposure to stress hormones in squirrels results in the faster growth rate of male offspring with a likely decrease in longevity [28]. It is possible that an increase in neurogenesis due to maternal separation in the present report is also part of a biphasic consequence of earlier stress that unfolds at a different trajectory than in males resulting in increasing neurogenesis during adulthood. Hippocampal neurogenesis has been previously reported to regulate anxiety and other stress-related behaviors [29], with enhanced neurogenesis being able to block anxiogenesis [30]. These observations point to a sexual dimorphism and likely protection of female rats from adverse effects of early-life stress through enhanced neurogenesis.

Most animal studies in behavioral neuroscience use male animals, with an assumption that hormonal variation in female represents a source of added experimental variation that should be avoided. However, our data support the notion that sex is an important variable, especially when studying the consequences of stress because differences in the hormonal landscape can moderate or extenuate plasticity brought about by the environment. Furthermore, the lack of diminishing effects of maternal separation in female rats presents an opportunity to build a model of emotional resilience.

## Declarations

### Author contribution statement

Yan Jun Lee: Performed the experiments; Analyzed and interpreted the data; Wrote the paper.

Amelia S. Koe, Archana Ashokan: Performed the experiments.

Rupshi Mitra: Conceived and designed the experiments; Analyzed and interpreted the data; Wrote the paper.

### Funding statement

This work was supported by the Ministry of Education, Singapore (# RG 46/12, to Rupshi Mitra).

### Competing interest statement

The authors declare no conflict of interest.

### Additional information

No additional information is available for this paper.
