# Supplementary material for: Female rats are resilient to the behavioral effects of maternal separation stress and exhibit stress-induced neurogenesis
Source: Heliyon. 2020 Aug 21;6(8):e04753. doi: 10.1016/j.heliyon.2020.e04753 (PMC7452405; doi:10.1016/j.heliyon.2020.e04753)
Supplement: Supplementary Data 1 [file mmc1.pdf]

Supplementary Data 1

Numerical values of data used in figures 1 through 3.

Data Depicted in Figure 1

% Open Arm Time		Emergence Latency (s)	
AFR	MS	AFR	MS
0.0	21.1	46.0	40.0
2.6	12.9	88.0	37.0
31.9	4.0	59.0	117.0
1.9	1.4	48.0	75.0
0.0	3.0	99.0	90.0
13.5	0.8	126.0	151.0
0.0	5.2	99.0	119.0
0.0	9.0	109.0	77.0
3.2	5.1	111.0	76.0
20.6	0.5	78.0	36.0
15.6	28.5	49.0	30.0
7.6	19.0	174.0	159.0
27.3	25.4	139.0	40.0
2.8	21.5	185.0	65.0
35.3	2.3	89.0	40.0
9.1	5.2	75.0	50.0
	10.9		63.0
	12.2		20.0
	2.2		198.0

Data Depicted in Figure 2

Total Dendritic Length (µm)		Total Number of Branches	
AFR	MS	AFR	MS
1764.4	2021.0	13.5	14.8
1886.6	1727.8	12.8	13.7
1633.7	1802.5	12.3	13.0
1950.2	2304.3	16.7	16.0
1512.4	1848.4	12.3	13.3
1662.3	984.9	13.0	10.5
1527.5	1360.8	11.3	12.0
1953.9	2095.1	17.3	17.5
1248.0	1466.4	9.0	10.8
1459.0	1500.8	12.2	10.3
	1636.0		14.3
	1897.6		12.8
	1547.2		12.0
	1263.4		14.0

Data Depicted in Figure 3

BrdU Positive Cells (Dorsal)	
AFR	MS
555.0	741.0
430.0	330.8
352.0	466.9
664.9	483.2
702.9	520.1
801.5	850.5
483.2	887.0
372.7	671.7
286.0	596.8
366.6	853.6
642.7	878.0
217.0	

BrdU Positive Cells (Ventral)	
AFR	MS
732.4	1072.2
437.2	622.4
398.0	412.5
591.0	419.0
477.2	495.4
830.4	1021.1
493.5	783.1
528.0	596.2
329.5	598.5
323.1	719.6
724.0	731.5
235.2	
